# Involvement of glycolysis/gluconeogenesis and signaling regulatory pathways in *Saccharomyces cerevisiae* biofilms during fermentation

**DOI:** 10.3389/fmicb.2015.00139

**Published:** 2015-02-23

**Authors:** Zhenjian Li, Yong Chen, Dong Liu, Nan Zhao, Hao Cheng, Hengfei Ren, Ting Guo, Huanqing Niu, Wei Zhuang, Jinglan Wu, Hanjie Ying

**Affiliations:** ^1^National Engineering Research Center for Biotechnology, College of Biotechnology and Pharmaceutical Engineering, Nanjing Tech UniversityNanjing, China; ^2^State Key Laboratory of Materials-Oriented Chemical Engineering, College of Biotechnology and Pharmaceutical Engineering, Nanjing Tech UniversityNanjing, China

**Keywords:** transcriptional profiles, *Saccharomyces cerevisiae*, biofilms, FLO family genes, biochemical pathways, signaling pathways, glycolysis/gluconeogenesis

## Abstract

Compared to free (free-living) cells, biofilm cells show increased resistance and stability to high-pressure fermentation conditions, although the reasons underlying these phenomena remain unclear. Here, we investigated biofilm formation with immobilized *Saccharomyces cerevisiae* cells grown on fiber surfaces during the process of ethanol fermentation. The development of biofilm colonies was visualized by fluorescent labeling and confocal microscopy. RNA from yeast cells at three different biofilm development periods was extracted and sequenced by high-throughput sequencing. We quantitated gene expression differences between biofilm cells and free cells and found that 2098, 1556, and 927 genes were significantly differentially expressed, respectively. We also validated the expression of previously reported genes and identified novel genes and pathways under the control of this system. Statistical analysis revealed that biofilm genes show significant gene expression changes principally in the initial period of biofilm formation compared to later periods. Carbohydrate metabolism, amino acid metabolism, signal transduction, and oxidoreductase activity were needed for biofilm formation. In contrast to previous findings, we observed some differential expression performances of FLO family genes, indicating that cell aggregation in our immobilized fermentation system was possibly independent of flocculation. Cyclic AMP-protein kinase A and mitogen-activated protein kinase pathways regulated signal transduction pathways during yeast biofilm formation. We found that carbohydrate metabolism, especially glycolysis/gluconeogenesis, played a key role in the development of *S. cerevisiae* biofilms. This work provides an important dataset for future studies aimed at gaining insight into the regulatory mechanisms of immobilized cells in biofilms, as well as for optimizing bioprocessing applications with *S. cerevisiae*.

## Introduction

Biofilms, as microbial communities, are dynamic environments wherein constituent cells propagate attached to biotic or abiotic surfaces (O'Toole et al., 2000; Hoyer, 2001; Reynolds and Fink, 2001). Microorganisms growing in biofilms live in a self-generated matrix of hydrated extracellular polymeric substances that form their immediate environment (Flemming and Wingender, 2010). It has become clear that biofilm-grown cells express properties distinct from free cells, one of which is an increased resistance to a variety of environmental stimulations (Mah and O'Toole, 2001).

The high antibiotic resistance of biofilms in the pathogenesis of some chronic human infections is now widely accepted. Biofilm formation had been described in prokaryotes such as *Pseudomonas aeruginosa*, *Staphylococcus aureus*, *Escherichia coli*, and the eukaryotic yeasts *Candida albicans* and *C. glabrata*, which have become major problems in immunosuppressed patients treated with broad-spectrum antibiotics (Hawser and Douglas, 1995; Stover et al., 2000; Boles and Horswill, 2008). To effectively remove pathogenic biofilms from medical devices, venous catheters, or urinary catheters *in vivo*, various methods were developed to characterize the relevant properties of biofilms. Several *in vitro* model systems have been developed to mimic biofilm growth occurring on infected medical devices. These models have provided the foundation for the investigating biofilm composition, architecture, and mechanisms of drug resistance (Baillie and Douglas, 2000; Chandra et al., 2001; Ramage et al., 2002). Transcription profiling experiments have identified potential biochemical pathways required for biofilm formation (García-Sánchez et al., 2004; Cao et al., 2005; Esteban et al., 2005). The structure and shear strength of microbial biofilms have been determined by confocal laser-scanning microscopy and fluid dynamic gaging using a novel rotating-disc biofilm reactor (Möhle et al., 2007). Some biochemical pathways, signaling regulatory mechanisms, and cell-cell interactions have been found in biofilms that were formed by pathogenic microorganisms (Dow et al., 2003; Goodman et al., 2004). Numerous investigators in the scientific community are actively investigating the biological processes that support biofilm formation with pathogenic microorganisms.

However, not all biofilms are harmful to humans, and biofilms have been applied widely in many fields. For example, in sewage treatment, biofilms growing on fibers facilitate the removal of organic waste products (Torregrossa et al., 2012), or may be used to biodegrade toxic chemicals (Cecie et al., 2012). Biofilm reactors were used in the laboratory of Prof. Shang-tang Yang (Ohio State University) to efficiently produce monoclonal antibodies and L(+)-lactic acid (Tay and Yang, 2002; Zhu and Yang, 2004). With a similar biofilm reactor, a high concentration of butyric acid (24.88 g·L^−1^) could be produced, which was higher than that produced by suspended-cell fermentation (13.70 g·L^−1^) (Jiang et al., 2010). In our lab, we have used immobilization technology to form biofilms on cotton fibers to efficiently produce various biochemical products, such as ethanol, butanol, and D-Lactic acid (Chen et al., 2013; Liu et al., 2014; Zhao et al., 2014). Compared to free culture cells, biofilm culture cells showed excellent tolerance to high concentrations of substrates and products, higher production efficiency, and shorter fermentation cycles. Therefore, biofilms can enhance many applications related to fermentation because of their excellent tolerances and/or stabilities. However, unlike pathogenic biofilms, the mechanism of superior growth and production during fermentation processes with *S. cerevisiae* biofilm remains unclear. A deeper understanding of biofilm formation in production processes will enable improvements in biofilm applications.

To better understand the mechanisms mediating biofilm formation, we performed comparative RNA-Seq analysis between *S. cerevisiae* biofilms and free cells to identify genes and biochemical metabolic pathways associated with biofilms during ethanol production. Statistical analysis revealed that genes involved in biofilms can show significantly gene-expression changes principally during the early period of biofilm formation, rather than in the maturation periods. We determined that the cyclic AMP-protein kinase A and mitogen-activated protein kinase pathways participate in biofilm formation, and some novel genes and previously implicated genes were validated. Furthermore, we also found that glycolysis/gluconeogenesis is necessary for *S. cerevisiae* biofilm formation.

## Materials and methods

### Yeast strains and growth conditions

*S. cerevisiae* 1308 (Chen et al., 2013) was stored in our laboratory and grown in conventional yeast extract peptone dextrose (YPD) growth medium (1% yeast extract, 2% peptone, and 2% glucose; solid media contained 2% agar) as described previously (Ito et al., 1983). The fermentation medium was optimized and contained 20% (5%) glucose, 0.4% peptone, 0.4% (NH_4_)_2_SO_4_, 0.3% yeast extract, 0.3% KH_2_PO_4_, 0.05% MgSO_4_, 0.005% ZnSO_4_·7H_2_O, and 0.005% FeSO_4_·7H_2_O. Yeast strains were grown overnight at 30°C in 250 mL Erlenmeyer flasks containing 30 ml YPD medium in a rotary shaker at 200 rpm/min. Ethanol fermentations were performed by adding 1 mL of overnight cultures to 250 mL Erlenmeyer flasks containing 100 mL fermentation medium, in the presence or absence of 5 g dry cotton fiber, on a rotary shaker at 250 rpm/min at 35°C. Thus, differences between the immobilized and free-fermentation reflected the presence or absence of cotton fibers in the fermentation, respectively. For immobilized culture, continuous batch fermentation was carried out; “the waste broth” was removed and fresh broth was added when the residual sugar was depleted (less than 1 g/L).

### Harvesting of cells and RNA isolation

Cells grown in biofilms were collected during three different biofilm growth stages, which are further defined in the Results and Discussion sections. Briefly, biofilm cells grown in the presence of cotton fibers were obtained at the biofilm attachment period (3 h), the sessile growth period (14 h), and the biofilm maturation period (30 h) and then washed twice in PBS (NaCl 0.8%, KCl 0.02%, Na_2_HPO_4_ 0.144%, KH_2_PO_4_ 0.024%, pH 7.4) to remove unattached free cells. Cotton fibers were placed into 80 mL PBS and treated with an ultrasonic cleaning device (Zips et al., 1990; Nishikawa et al., 2010) and vortex instrument. Subsequently, the biofilm cells were collected by centrifugation (5000 rpm, 2 min). Cell pellets were immediately frozen in liquid nitrogen and stored at −80°C. Free-culture cells were collected after 2.5 h of growth by centrifugation (5000 rpm, 2 min) and were washed twice in PBS. Cell pellets were immediately frozen in liquid nitrogen and stored at −80°C. Three samples of the same stages were pooled and homogenized, and total RNA was extracted to make staged samples for transcript analyses (Irie and Kuratani, 2011). To improve the reliability of data in each developmental stage, we took 2 G of sequencing data. RNA was isolated from *S. cerevisiae* free cells or biofilm cells as described previously (Ying et al., 2005).

### cDNA library construction, illumina sequencing, and *de novo* assembly

The experiment methods (Ren et al., 2012) of Ren were referenced in this section. Magnetic beads (Illumina) with Oligo (dT) were used to isolate poly (A) mRNA from total RNA, which was treated with DNase I. Purified mRNA was fragmented (200–700 nt) and used as templates for the first-strand cDNA synthesis by random hexamer-primers. The second-strand cDNA was synthesized using buffer, dNTPs, RNase H, and DNA polymerase I. The resulting short double-stranded cDNA fragments were purified with a QIAquick PCR extraction kit (vendor) and dissolved in EB buffer (10 mM Tris-Cl, pH 8.5) for end reparation and the addition of an “A” base. Subsequently, cDNA fragments were ligated to Illumina sequencing adapters and purified by agarose gel electrophoresis. Suitably sized fragments (200 ± 25 bp) were then selected as templates for PCR amplification. The Agilent 2100 Bioanalyzer and ABI StepOnePlus Real-Time PCR System were used during quality control (QC) testing for quantification and qualification of the sample library. Primary sequencing data (raw reads) produced by the Illumina HiSeq™ 2000 were subjected to QC tests to determine if resequencing was necessary. The QC process involved the removal of adapter reads, reads with >5% unknown bases, and reads with >30% low-quality bases (i.e., with sequencing quality ≤10). Following QC analysis, raw reads were filtered and clean reads were aligned with reference sequences using SOAPaligner/SOAP2 software (Ruiqiang et al., 2009). Alignment data were utilized to calculate read distributions for reference genes and to perform coverage analysis. RNA-Seq experiments facilitated the downstream analyses of gene expression levels and then differential expression analysis. We also performed Gene Ontology (GO)-enrichment analysis and pathway enrichment analysis.

### RNA-seq data normalization and statistical analysis of gene expression

The expression level for a given gene of interest (“gene A”) was calculated by the reads per kilobase transcriptome per million mapped reads (RPKM) method (Mortazavi et al., 2008), using the following formula:

$$RPKM=\frac{10^{6}C}{NL/10^{3}},$$

where *C* is the number of reads that are uniquely aligned with gene A, *N* is the total number of reads that are uniquely aligned to all genes, and *L* is the number of bases aligned with gene A.

To determine the significance of digital gene expression profiles, we first denoted the number of unambiguous clean tags (i.e., RNA-Seq reads) for gene A as x. Given that each gene's expression occupies only a small part of the overall library, x yields to the Poisson distribution:

$$p\left(x\right)=\frac{e^{-\lambda }\lambda^{x}}{x!},$$

where λ is the actual (albeit unknown) number of transcripts of this type per *N* clones in the library (Audic and Claverie, 1997).

The total number of clean tags of sample 1 was designated as *N*_1_, while the total number of clean tags for sample 2 was designated as *N*_2_; thus, gene A holds x tags in sample 1 and y tags in sample 2. The probability of gene A being expressed equally between two samples can be calculated using the following formula:

$$\begin{array}{l} \;2\sum_{i\;=\;0}^{i\;-\;y}p\left(i|x\right)\;\\ \text{or }2\times \left(1-\sum_{i\;=\;0}^{i\;-\;y}p\left(i|x\right)\right)\left(\text{if }\sum_{i\;=\;0}^{i\;-\;y}p\left(i|x\right)>0.5\right)\\ \;p\left(\text{y}|\text{x}\right)={\left(\frac{N_{2}}{N_{1}}\right)}^{y}\frac{\left(\text{x}+\text{y}\right)!}{x!y!{\left(1+\frac{N_{2}}{N_{1}}\right)}^{\left(\text{x }+\text{ y }+\;1\right)}} \end{array}$$

*P*-value determinations were used for differentially expressed gene analysis. Because differentially expressed gene analysis tests thousands of hypotheses simultaneously (in determining whether given genes are differentially expressed between two groups), corrections for false positives (type I errors) and false negatives (type II) errors were performed using the false discovery rate (FDR) method (Benjamini and Yekutieli, 2001). Thus, for example, if it is desired that the error ratio stays below a cutoff (e.g., 5%), then the FDR should be preset to a number ≤0.05 (Benjamini and Yekutieli, 2001). In this study, we set FDR ≤ 0.001 and the absolute value of Log_2_ Ratio ≥ 1 as criteria for assessing the significance of differential gene expression. GO enrichment analysis and pathway enrichment analysis were also performed.

### Real-time quantitative PCR analysis

RNA extractions and quality control experiments were performed as described in the previous section. Reverse transcription was performed using the AMV First Strand cDNA Synthesis Kit (Sangon Biotech), according to the manufacturer's instructions. Primer 5 software was used to select the primers. Quantitative real-time PCR assays were performed with the SYBR Green PCR Master Mix (Applied Biosystems) in a StepOnePlus Real-Time PCR System. Reactions were performed according to the manufacturer's instructions, and three technical replicates were included for each sample. Gene transcription levels was determined according to the 2^−ΔΔCT^ method, using the 18S rRNA gene as a reference gene for normalizing gene expression levels, as described (Stevenson and Weimer, 2005). To verify RNA-Seq data, Pearson correlation coefficient values were calculated using Microsoft Excel (Microsoft Corporation, Redmond, WA, USA), and used as an indicator for the degree of correlation for the compared pairs.

### Scanning electron microscopy (SEM) analysis

Biofilm cells and free cells were harvested at the same time points mentioned in the “Harvesting of cells and RNA isolation” section, washed twice with PBS buffer, and stored at −80°C. Free cells and biofilm cells were dried using a FreeZone® 4.5 Liter Freeze Dry System (Labconco, Kansas City, MO, USA) and sputter-coated with gold. Images were obtained using a Hitachi S-4800 field emission SEM.

### Confocal laser scanning microscopy (CLSM) analysis

Like SEM analysis, biofilm cells and free cells were harvested, washed twice with PBS buffer and stained immediately with FUN-1 and Alexa Fluor 488-conjugated ConA (both from Molecular Probes, Inc., Eugene, OR). FUN-1 (excitation wavelength: 543 nm; emission wavelength: 560 nm) is converted to an orange-red molecule in metabolically active cells, while Alexa-Fluor 488-conjugated ConA (excitation wavelength: 488 nm; emission wavelength: 505 nm) binds to the glucose and mannose residues of cell-wall polysaccharides with green fluorescence (Chandra et al., 2001). Confocal images were captured using a Leica TCS SP5 II.

### Metabolite analyses and calculations

The 0.5 mL samples were sampled every 2 h from fermentation systems, centrifuged (5000 rpm, 2 min) and pasted the organic membrane. Glucose and ethanol concentrations were determined by high-performance liquid chromatography analysis (Agilent 1200 infinity series; Hewlett–Packard, CA, USA), using an Aminex HPX-87H ion-exclusion column (300 × 7.8 mm; Bio-Rad Laboratories, Hercules, CA, USA) heated to 25°C. The analytes were separated with a mobile phase of 5.0 mM H_2_SO_4_ at a flow rate of 0.6 mL/min and detected using a refractive index detector. Cell concentrations were determined spectrophotometrically as the OD_600_nm.

## Results and discussion

### Temporal characterization of *S. cerevisiae* biofilms

**Figure 1:**
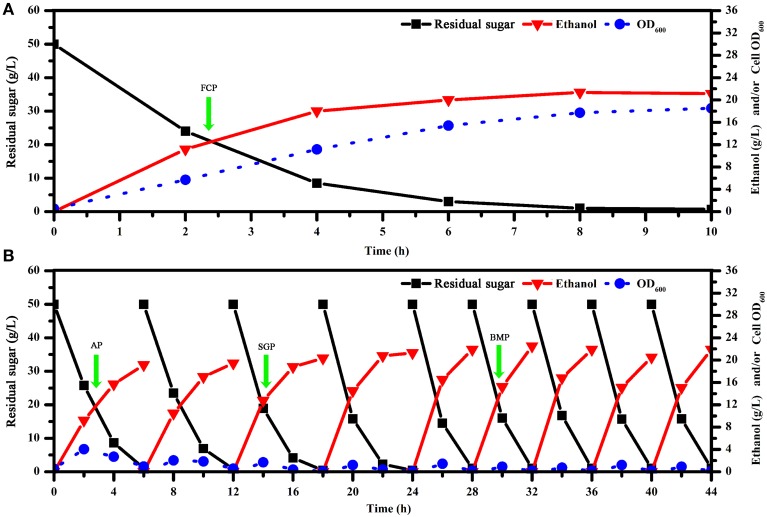
**Kinetics of batch fermentation with free and immobilized cell cultures. (A)** Kinetics of fermentation in free cell cultures. **(B)** Kinetics of batch fermentation in immobilized cultures. To reduce the impact of cell-growth conditions over time on gene expression, we selected optimal sampling periods with maximal consistency in terms of residual sugar and ethanol concentrations that were also representative of biofilm development processes. OD, optical density; FCP, free cell period; AP, attachment period; SGP, sessile-growth period; BMP, biofilm-maturation period.

**Figure 2:**
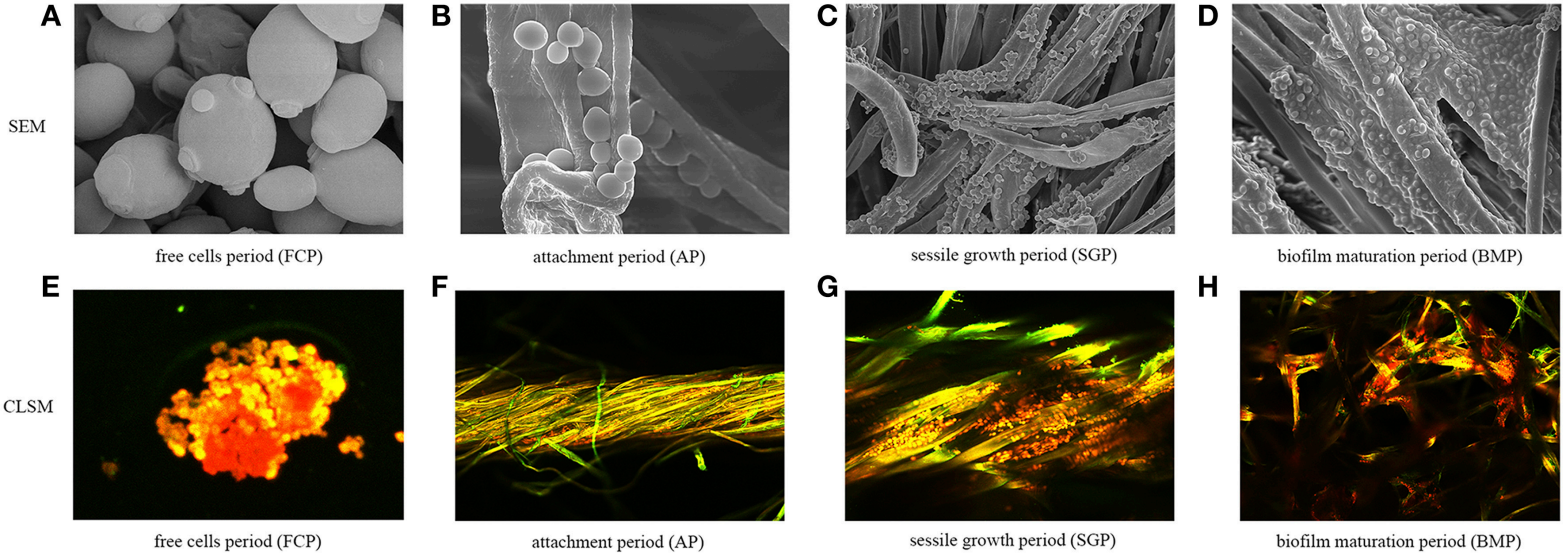
**Temporal biofilm characterization at different periods of biofilm development. (A)** SEM images of free yeast cells, used as control throughout this study. **(B–D)** SEM images of biofilms taken at three different growth periods. **(E)** CLSM images of free cells. **(F–H)** CLSM images of biofilms taken at three different growth periods. Strains were dyed orange-red by FUN-1 and polysaccharides were dyed green by Alexa Fluor 488-conjugated ConA.

In batch fermentations with immobilized cells, we found that immobilized fermentation required a shorter fermentation cycle and had higher fermentation efficiency compared to traditional suspension fermentation (Figure 1). As demonstrated previously, biofilms formed by immobilized cells might play a key role in such systems (Chen et al., 2013; Liu et al., 2014; Zhao et al., 2014). We also found that successive fermentation cycles became shorter and fermentation efficiency increased through the fifth batch, in which fermentation parameters were held steady (Figure 1). Quantitative and qualitative analysis of biofilms showed that *S. cerevisiae* biofilm development was a process that could be characterized temporally (Figure 2). After considering the characteristics of the immobilized fermentation process and images of biofilms obtained over time, we categorized biofilm development in three developmental periods: the attachment period, sessile-growth period, and the biofilm-maturation period (Nett et al., 2009).

Scanning electron microscopy and confocal microscopy were employed to characterize the physiological characteristics of biofilms. The images demonstrated a temporal continuity during biofilm formation (Figure 2). Temporal characterization of colonization revealed that a sessile-growth period was required for a mature biofilm to develop (Figures 2C,G). During the attachment period, individual cells adhered directly to the fiber surface, as did microcolonies, composed of clumps of cells. At the biofilm-maturation period, biofilms that were sticky in appearance largely covered the fiber surface and appeared to reach a maximum density, after which no further increase occurred. However, even within mature biofilms, colonization was patchy, with some areas being densely populated by various layers of cells and other areas showing few adherent cells. These patterns were likely caused by the continuous shedding of dead cells from biofilms, resulting in vacant areas that could potentially be recolonized subsequently. To further study biofilm formation at the molecular level, yeast RNAs were extracted from immobilized cells in different stages of biofilm development or from free cells and were then analyzed by the high-throughput sequencing.

### Gene expression differences between biofilm cells and free cells

**Figure 3:**
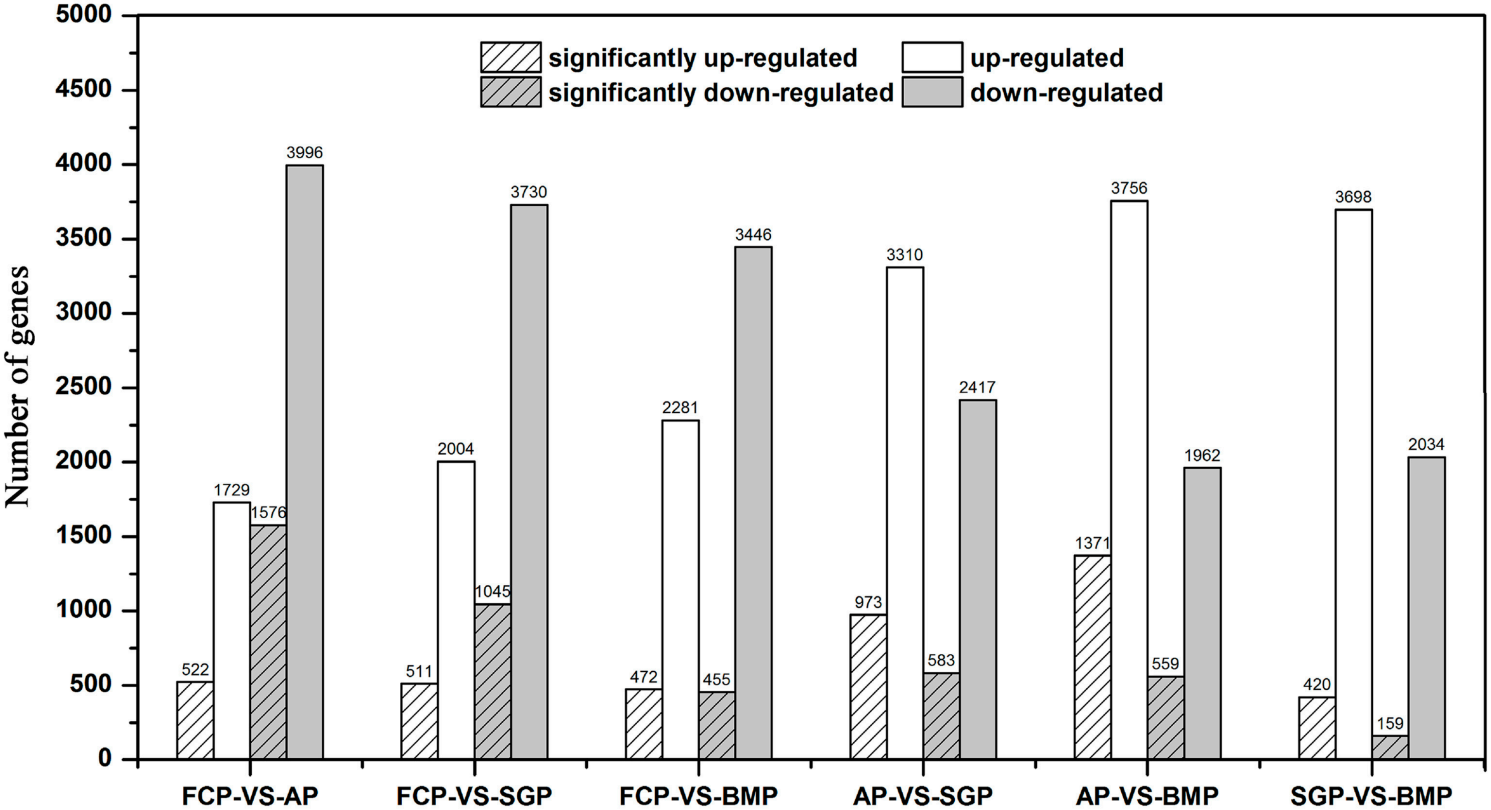
**Gene-expression differences under differing growth conditions**. White bars represent the number of up-regulated genes, and gray bars represent the number of down-regulated genes. Hatched bars represent the number of differentially expressed genes whose expression levels were significantly different from those observed in control cells (FDR ≤ 0.001, log2 Ratio ≥ 1). FCP, free cells period; AP, attachment period; SGP, sessile-growth period; BMP, biofilm-maturation period.

mRNA fractions purified from *S. cerevisiae* cells growing in the attachment period, the sessile-growth period, and the biofilm-maturation period were analyzed to determine their respective gene expression profiles and to identify differentially expressed genes. When comparing gene expression levels in free cells vs. cells harvested in the attachment period, 5725 genes were differentially expressed, of which 1576 were significantly down-regulated and 522 were significantly up-regulated (Figure 3). In yeast cells harvested during the sessile-growth period, compared with freely growing cells, 1556 genes were significantly differentially expressed, of which 1045 were significantly down-regulated and 511 were significantly up-regulated. Similarly, 455 genes were significantly down-regulated during the biofilm-maturation period, and 472 genes were significantly up-regulated, compared with freely growing cells. Interestingly, the number of down-regulated genes in the free cells vs. the biofilm cells in the attachment period was 3-fold higher than the number of up-regulated genes, a higher ratio than observed in free cells vs. sessile-growth period cells, or free cells vs. biofilm-maturation period cells. *S. cerevisiae* cells began to adhere to the fiber surface during the attachment period (Figure 2); thus, some differentially expressed genes may have been involved in adapting to the changing environment. A reasonable possibility was that intracellular signaling pathways were activated in microorganisms while they interacted with the media surface and initiated biofilm development. Results from a previous study indicated that the adherence to media surfaces may be caused by specific interactions with a microorganism and that such interactions are necessary to initiate biofilm formation (Prouty et al., 2002). Analysis of our gene expression data revealed that the total number of significantly up-regulated genes changed only slightly during the sequential steps of the biofilm development process. However, the number of the significantly down-regulated genes decreased markedly. These observations raise the possibility that some parts of genes, particularly those that were significantly up-regulated, may be stably expressed during successive stages of biofilm development and may be necessary for biofilm maintenance. The trend toward decreasing numbers of significantly down-regulated genes during progressive biofilm development may reflect a redirection of cellular activities away from propagation or biofilm formation and toward alternative metabolic pathways, because biofilms appeared to reach a maximum density, after which no further increase occurred at the mature biofilm period (Frese et al., 2013).

### Differential mRNA expression patterns of cells grown in attachment period, the sessile-growth period, and the biofilm-maturation period

Moreover, gene expression patterns in attachment period vs. sessile-growth period cells, attachment period vs. biofilm-maturation period cells, and sessile-growth period cells vs. biofilm-maturation period cells were compared to identify differentially expressed transcripts. Down-regulated and up-regulated genes were identified based on differences for which *p*-values were less than 0.001. A total of 973, 1371, and 420 genes were significantly up-regulated, respectively in attachment period vs. sessile-growth period cells, attachment period vs. biofilm-maturation period cells, and sessile-growth period vs. biofilm-maturation period cells. In addition, 583, 559, and 159 genes were significantly down-regulated, respectively (Figure 3). As expected, these comparisons indicated the expression levels of many genes were enhanced, and obvious differences were observed during biofilm development.

### Gene ontology (GO) functions and KEGG pathway analysis

**Figure 4:**
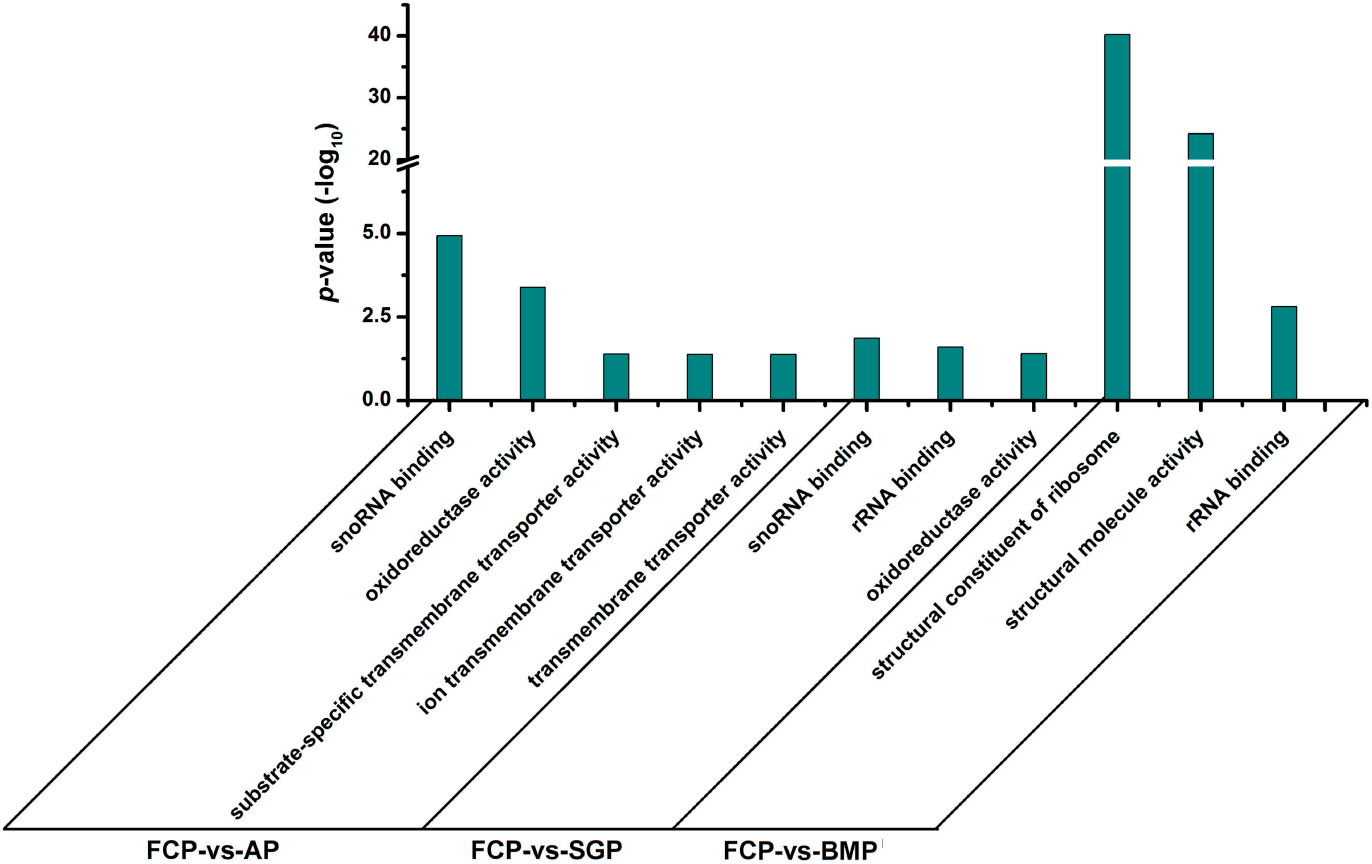
**Bar chart of enrichment ratios for Gene Ontology function categories**. Gene expression levels of cells grown in the free cell period (FCP) were set as the background level. Corrected *p*-value ≤ 0.05 was used as a threshold. GO terms fulfilling this condition are defined as significantly enriched GO terms for the differentially expressed genes. AP, attachment period; SGP, sessile-growth period; BMP, biofilm-maturation period.

GO enrichment analysis was performed with the differentially expressed genes identified by RNA-Seq analysis, and all of cellular components, molecular functions, and biological processes are shown in Supplementary Tables S1–S3. We focused on molecular functions to identify gene categories that are potentially associated with biofilms (Figure 4). Genes involved in oxidoreductase and transmembrane transporter activity were found to be differentially expressed when comparing free cells vs. attachment-period cells and free cell vs. sessile-growth period cells. However, some structural constituents and molecules were produced at elevated levels in free cell vs. biofilm-maturation period cells. Functions related to RNA binding showed sustained elevation during the entire course of biofilm development.

KEGG pathway analysis (classification criteria based on KEGG pathways: http://www.genome.jp/kegg/pathway.html) was also used to identify significantly enriched metabolic or signal transduction pathways associated with differentially expressed genes in biofilms, in comparison with expression profiles observed in free cells. The KEGG pathways controlled by differentially expressed genes at different periods of biofilm development are listed in Supplementary Tables S4–S6. Data analysis highlighted gene expression differences (*Q*-values < 0.05) occurring during biofilm development relating to several keys pathways (Table 1). These pathways were mainly involved in carbohydrate, energy, amino acid, lipid metabolism, and transcription/translation. These early events increased the intracellular pools of carbohydrates, amino acids, and lipids, thereby facilitating preparation for biomass increases characteristic of the sessile-growth period. During the biofilm-maturation period, only three pathways were significantly enriched, indicating that a diminution of metabolic differences may have occurred by that point in development. Numerous pathways were activated in the initial phases of biofilm formation, which may also have resulted from interaction between cells and solid surfaces. One hypothesis to explain these results is that cell surface signaling molecules in yeast can respond to changes in the external environment by launching a series of regulatory mechanisms, such as the mitogen-activated protein kinase, high osmolarity glycerol, and Ras-Pka pathways, which can either stimulate or repress pathways during biofilm formation (O'Toole and Kolter, 1998; Li et al., 2002).

**Table 1:** **Overview of significantly enriched KEGG-pathways in biofilms**.

**COMPARISON OF THE FREE CELL PERIOD (FCP) vs. THE ATTACHMENT PERIOD (AP)**					
**Pathway ID**	**Pathway**	**DEGs with pathway annotation (648)**	**All genes with pathway annotation (2143)**	***P*-value**	*Q*-value^*^
ko00020	Citrate cycle (TCA cycle)	26 (4.01%)	32 (1.49%)	2.70E-09	2.53E-07
ko00010	Glycolysis/ Gluconeogenesis	37 (5.71%)	55 (2.57%)	1.05E-08	4.93E-07
ko03008	Ribosome biogenesis in eukaryotes	50 (7.72%)	91 (4.25%)	4.66E-07	1.46E-05
ko00190	Oxidative phosphorylation	42 (6.48%)	76 (3.55%)	3.32E-06	7.81E-05
ko00630	Glyoxylate and dicarboxylate metabolism	17 (2.62%)	22 (1.03%)	6.50E-06	1.22E-04
ko00620	Pyruvate metabolism	22 (3.4%)	33 (1.54%)	1.47E-05	2.31E-04
ko00380	Tryptophan metabolism	13 (2.01%)	16 (0.75%)	3.44E-05	4.63E-04
ko00680	Methane metabolism	19 (2.93%)	28 (1.31%)	3.99E-05	4.69E-04
ko00071	Fatty acid metabolism	13 (2.01%)	17 (0.79%)	0.000106059	1.11E-03
ko00260	Glycine, serine, and threonine metabolism	19 (2.93%)	30 (1.4%)	0.000164023	1.54E-03
ko03020	RNA polymerase	18 (2.78%)	30 (1.4%)	0.000638511	5.30E-03
ko00920	Sulfur metabolism	12 (1.85%)	17 (0.79%)	0.000676888	5.30E-03
ko00030	Pentose phosphate pathway	17 (2.62%)	28 (1.31%)	0.000748489	5.41E-03
ko00230	Purine metabolism	43 (6.64%)	94 (4.39%)	0.000856499	5.75E-03
ko00640	Propanoate metabolism	9 (1.39%)	12 (0.56%)	0.001749713	1.10E-02
**COMPARISON OF THE FREE CELL PERIOD (FCP) vs. THE SESSILE GROWTH PERIOD (SGP)**					
**Pathway ID**	**Pathway**	**DEGs with pathway annotation (518)**	**All genes with pathway annotation (2143)**	***P*-value**	*Q*-value^*^
ko03010	Ribosome	69 (13.32%)	183 (8.54%)	1.33E-05	0.001223771
ko00640	Propanoate metabolism	9 (1.74%)	12 (0.56%)	0.000285205	0.013119444
ko00900	Terpenoid backbone biosynthesis	11 (2.12%)	18 (0.84%)	0.000872115	0.023510158
ko00380	Tryptophan metabolism	10 (1.93%)	16 (0.75%)	0.001190103	0.023510158
ko00230	Purine metabolism	36 (6.95%)	94 (4.39%)	0.001277726	0.023510158
ko00100	Steroid biosynthesis	9 (1.74%)	14 (0.65%)	0.00160835	0.024661367
ko00040	Pentose and glucuronate interconversions	7 (1.35%)	10 (0.47%)	0.002781217	0.036553138
ko00650	Butanoate metabolism	10 (1.93%)	18 (0.84%)	0.004022897	0.043771998
ko00561	Glycerolipid metabolism	11 (2.12%)	21 (0.98%)	0.004678847	0.043771998
ko03008	Ribosome biogenesis in eukaryotes	33 (6.37%)	91 (4.25%)	0.005600554	0.043771998
ko00260	Glycine, serine, and threonine metabolism	14 (2.7%)	30 (1.4%)	0.005637695	0.043771998
ko00620	Pyruvate metabolism	15 (2.9%)	33 (1.54%)	0.005709391	0.043771998
**COMPARISON OF THE FREE CELL PERIOD (FCP) vs. THE BIOFILM MATURATION PERIOD (BMP)**					
**Pathway ID**	**Pathway**	**DEGs with pathway annotation (376)**	**All genes with pathway annotation (2143)**	***P*-value**	*Q*-value^*^
ko03010	Ribosome	120 (31.91%)	183 (8.54%)	5.17E-53	4.55E-51
ko00640	Propanoate metabolism	8 (2.13%)	12 (0.56%)	0.000215299	9.47E-03
ko00010	Glycolysis/ Gluconeogenesis	19 (5.05%)	55 (2.57%)	0.00161897	4.75E-02

* *Pathways with Q-values ≤ 0.05 were significantly enriched in differentially expressed genes (DEGs)*.

### Expression of genes related to flocculation and signaling

Yeast flocculation is a common, reversible, and calcium-dependent process, in which cells adhere to form flocculates consisting of thousands of aggregated cells (Stratford, 1989; Bony et al., 1997). Cell–cell adhesion is a highly complex process that is affected by various environmental factors and is regulated by specific flocculation-associated genes. In yeast, the FLO family of genes is similar to ALS family in *C. albicans* and includes *Flo1*, *Flo5*, *Flo9*, *Flo10*, and *Flo11* (Teunissen and Steensma, 1995). The Flo1, Flo5, Flo9, and Flo10 proteins (known as flocculins) promote cell-cell adhesion and assimilate free-floating yeast cells into colonies (Goossens and Willaert, 2010). The *Flo11* gene encodes a widely studied GPI-anchored cell-surface flocculin glycoprotein that is critical in regulating yeast biofilm formation through a large complex network (Lo and Dranginis, 1998; Andersen et al., 2014). In our RNA-Seq data, *Flo11* gene expression levels in the attachment, sessile-growth, and biofilm-maturation periods of biofilm development increased by 6.8-fold, 5.0-fold, 18.4-fold, respectively, compared with the expression levels observed in free cells. Similar observations were also reported previously (Zara et al., 2012). However, the other FLO family genes mentioned above were all down-regulated in relation to free cells in our biofilm system, in contrast to previous reports (Bester et al., 2006; Tofalo et al., 2014). *Bsc1* shows sequence similarity to cell-surface flocculin gene *Flo11* and was down-regulated 42-fold, 4-fold, and 3-fold, respectively, which was opposite to the expression profile observed for *Flo11*. This result implied that cell aggregation in our immobilized fermentation was possibly independent of flocculation, a discovery with important implications for understanding the mechanism of biofilm formation by immobilized cells.

**Figure 5:**
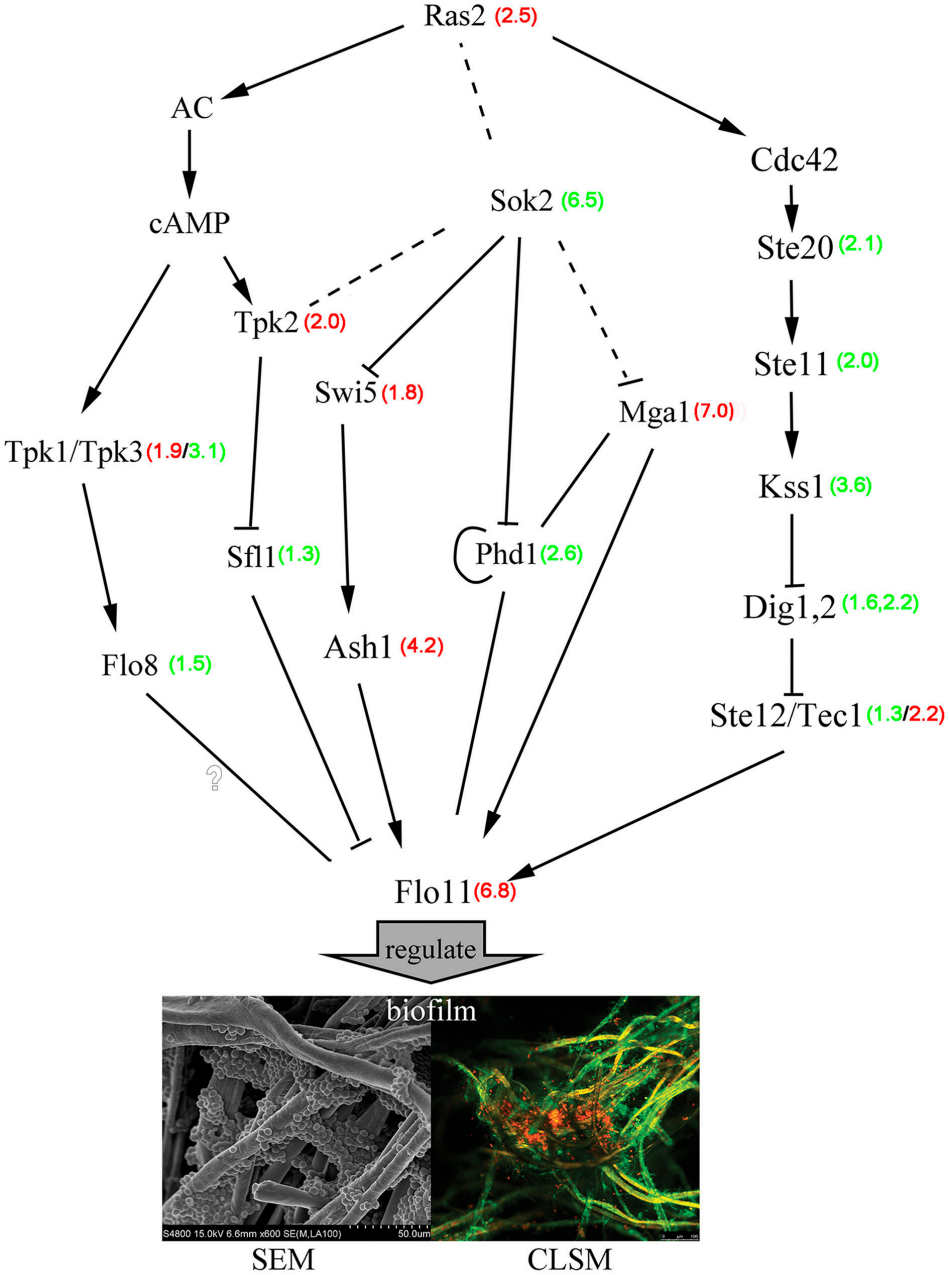
**Hypothetical gene regulation and signaling control in *S. cerevisiae* biofilms**. Red numbers represent the degree of gene up-regulation compared with free cells, and green numbers represent the degree of gene down-regulation compared with free cells. The full lines represented the probable regulation directly and the broken lines represented the probable regulation indirectly.

Importantly, several signaling cascades including the Ras/cAMP-protein kinase A (PKA) and MAPK-dependent filamentous growth pathways tightly control the synthesis of different adhesins through some FLO genes (Verstrepen and Klis, 2006; Valle et al., 2013). Analysis of differential gene expression revealed a large set of genes that were activated during the initial phase of biofilm formation, thus, we focused on genes' important for the biofilm at attachment period. In the MAPK pathway, the transcription factor *Tec1*, which can transactivate flocculent gene expression, was expressed at 2-fold higher levels than those found in free cells. Homozygous *Tec1*-knockout yeast cells showed markedly impaired biofilm development (Nobile et al., 2006). However, *Ste12*, which cooperates with the *Tec1* transcription factor to regulate genes specific for invasive growth, was marginally down-regulated. We observed a 3-fold down-regulation of the *Kss1* (Mitogen-activated protein kinase), which can reverse the Dig1,2-mediated inhibition to the MAPK pathway proteins Ste12/Tec1. Furthermore, the MAPK pathway genes *Ste20* and *Ste11* were also down-regulated in biofilm cells. The cAMP-PKA pathway members *Tpk3* and *Flo8* are required for biofilm formation and were down-regulated when grown under low-glucose conditions (Andersen et al., 2014). We also found that expression of the transcriptional activator genes *Phd1*, *Ash1*, and *Mga1* and of the nuclear protein *Sok2* gene significantly differed in biofilm cells during the attachment period. *Mga1* and *Ash1* regulate *Flo11* expression, which is required for filamentous growth, and are also required for flocculation in *sok2/sok2* mutant strains (Pan and Heitman, 2000). *Phd1* transcription is apparently controlled by several regulators of filamentous growth (Sok2, Mga1, and Phd1 itself), which bind to the *Phd1* promoter region, and *Phd1* activity is regulated by the cAMP-PKA pathway (Borneman et al., 2006). *Phd1* was down-regulated in our biofilm cells, in contrast to findings from a previous study (Pan and Heitman, 2000). *Efg1*, the *C. albicans* homolog of *S. cerevisiae Phd1*, was also markedly down-regulated in the presence of low glucose and is an essential regulator of morphogenesis, cell wall remodeling, and virulence of *C. albicans* (Leng et al., 2001; Sohn et al., 2003). These observations clearly show that the MAPK and cAMP-PKA pathways participated in biofilm formation. Additional genes participated in the regulatory processes associated with the MAPK and cAMP-PKA pathways. Hypothetical regulatory networks are represented schematically in Figure 5 to provide a potential regulatory mechanism for biofilm formation.

### Glycolysis/gluconeogenesis pathways and *S. cerevisiae* biofilm formation

**Figure 6:**
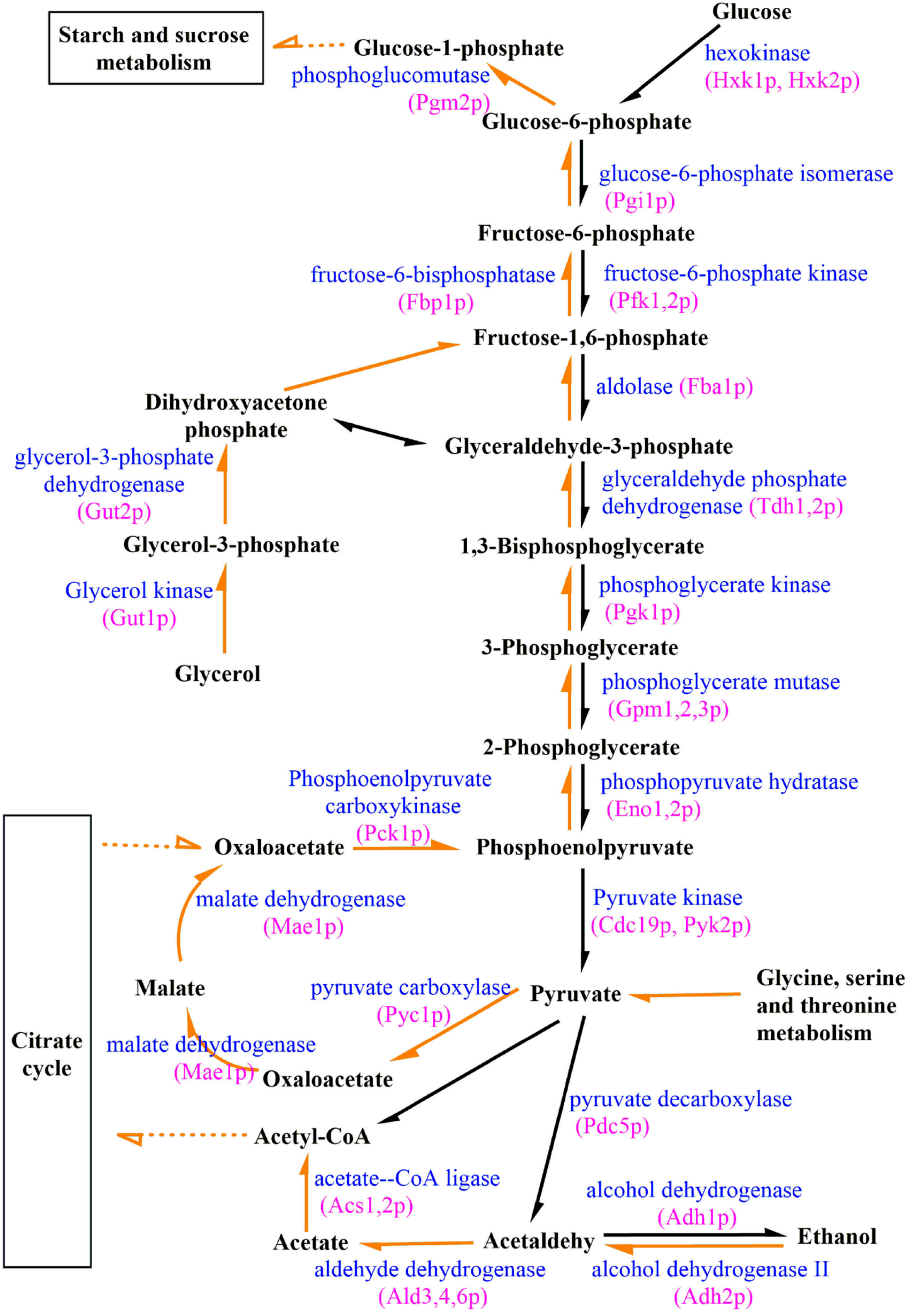
**Differential expression of genes involved in glycolysis/gluconeogenesis during biofilm growth**. Orange arrows represent genes involved in gluconeogenesis, and black arrows represent genes involved in glycolysis. The full lines represented the regulation directly and the broken lines represented the regulation indirectly.

Because carbohydrates serve as major constituents of biofilms (Lal et al., 2010), studying the expression of genes regulating their production by RNA-Seq may provide insights into the mechanisms underlying biofilm genesis. Compared with expression levels observed in free cells, pathways related to carbohydrate metabolisms (Table 1) were significantly enriched in biofilm cells, as demonstrated by a KEGG database search. Further comparative analysis showed that key genes regulating gluconeogenesis, such as *Fbp1* and *Pck1*, were up-regulated 239-fold and 555-fold, respectively in attachment-period cell vs. free cells. However, this change was not observed when comparing free cells with sessile-growth period or biofilm-maturation period cells. Visualization and gene expression analysis of biofilms also suggested that genes encoding enzymes involved in carbohydrate synthesis were differentially regulated during biofilm growth. Therefore, we investigated potential correlations between carbohydrate synthesis and *S. cerevisiae* biofilms to characterize biofilm formation. By analyzing transcription data and KEGG metabolism maps (Figure 6), we discovered that the *Pyc1*, *Pyc2* and *Mae1* genes, which encode pyruvate carboxylase (*Pyc1* and *Pyc2*) or malate dehydrogenase (*Mae1*), were up-regulated. Some genes (i.e., *Agx1*, *Gcv1, Gcv2, Gcv3*, *Cha1*, or *Ilv1*), encoding glucogenic amino acids such as glycine, serine, or threonine were regulated to promote gluconeogenesis. Gut1, a glycerol kinase that converts glycerol to glycerol-3-phosphate, was up-regulated by 6.6-fold, 1.4-fold, and 2.3-fold during the sequential periods of biofilm development. Thus, glycerol may be used as starting material in metabolic pathways mediating carbohydrate synthesis. To more accurately and comprehensively understand the role(s) of gluconeogenesis in biofilm development, we also analyzed the expression levels of genes involved in glucose and glycogen synthesis/utilization. We found that genes related to glucogenesis and trehalose biosynthesis were up-regulated in biofilms and that dextran-associated genes were suppressed. *Tps1*, which encoding trehalose-6-phosphate synthase 1 (a key enzyme for trehalose biosynthesis), and the phosphoglucomutase genes *Pgm1* and *Pgm2* were expressed with biofilm formation. Recently, several studies have shown that trehalose, beyond its primary roles in the carbohydrates, plays an important role in biofilm formation and protecting yeast against a variety of stresses (Li et al., 2012; Zhu et al., 2012). The above analyses showed that gluconeogenesis-pathway genes were activated during early biofilm development. Some genes involved in glucogenic amino acid production and glycerol degradation were up-regulated, and glucogenesis and trehalose biosynthesis were enhanced during the attachment period.

To explore whether enhanced gluconeogenesis can influence ethanol production in the initial biofilm formation period, we analyzed gene expression levels related to glycolysis at the three successive periods of biofilm development. Our data showed that the expression of all genes involved in glycolysis were suppressed in attachment-period cells compared to free cells (Figure 6). During the progressive development of biofilms, suppression of glycolysis-pathway gene expression was alleviated, and some genes were positively regulated during the biofilm-maturation period. The *Pdc5* gene, which encodes pyruvate decarboxylase, a key enzyme in alcoholic fermentation and the alcohol dehydrogenase genes *Adh1*, *Adh4*, *Adh5*, and *Adh6* were down-regulated at the attachment period. However, at the sessile-growth and biofilm-maturation periods, suppressions of these genes were reversed. We found that the expression of glycolysis genes were inversely correlated with those of gluconeogenesis genes, suggesting that biofilm formation resulted from suppressed glycolysis and enhanced gluconeogenesis during the attachment period. As biofilms mature, ethanol production through glycolysis can predominate. In addition, the role of some genes in biofilm formation, such as *Ald3*, *Ald4*, and *Ald6*, remains unclear. Therefore, further studies are needed to verify the involvement and characterize the functional roles of some genes during biofilm formation with *S. cerevisiae*.

### Amino acid metabolism and energy production

We found that the metabolisms of various amino acids, including glycine, serine, threonine, tryptophan, valine, leucine, and isoleucine, were dysregulated during the entire process of biofilm development, compared to free cells. These results are consistent with those previous transcriptional analysis studies, which revealed that genes involved in amino acid biosynthesis were up-regulated in *C. albicans* biofilms compared to planktonic cells (Zhu et al., 2012). In addition, the importance of amino acid biosynthesis during biofilm formation has been described previously (García-Sánchez et al., 2004). *Cys3* and *Cys4*, encoding sulfur amino acid, were up-regulated during biofilm development. In addition, some citrate cycle (tri-carboxylic acid; TCA) genes were down-regulated during biofilm development, resulting in the acceleration of amino acid accumulation. Similar results were reported in a previous study (Zhu et al., 2012). Some energy-metabolism pathways such as oxidative phosphorylation and sulfur metabolism were enriched in biofilms compared with free cells, suggesting that electron transport was required to biofilm formation.

### Transcription factors and resistance genes

We validated the correlation of previously implicated genes with biofilm development and identified novel genes under control of this system. *Hsp12*, a plasma membrane protein regulated by the high osmolarity glycerol and Ras-Pka pathways (Varela et al., 1995), was up-regulated by 181-fold, 49-fold and 13-fold, respectively during the sequential biofilm periods. This gene was induced by heat shock, oxidative stress, osmostress, stationary phase, glucose depletion, and alcohol (Dueñas-Sánchez et al., 2012). The analogous gene *Gre1* was also up-regulated during the attachment and sessile-growth periods. Similarly, *Sip4*, a C6 zinc cluster transcriptional activator that positively regulates gluconeogenesis, was up-regulated in early biofilms. In contrast, *Mig1*, a transcription factor related to glucose repression (Klein et al., 1998), was down-regulated 26-fold during the attachment period in biofilms. The levels of transcription of some key genes in RNA-Seq, such as *Flo11*, *Pck1*, *Fbp1*, *Hsp12*, *Mig1*, and *Sip4*, were verified by RT-PCR in this study (Supplementary Table S7). Most of the RT-PCR data matched the RNA-Seq based the values (fold changes of gene expression in biofilm cells vs. free cells) with a correlation coefficient of 0.95 for the set of six selected genes, which indicated that our RNA-Seq result is accurate and the conclusion from RNA-Seq should be reliable. However, the regulatory mechanisms of these genes in biofilms were non-determined and should to be investigated in future studies.

## Conclusions

Here, we analyzed gene expression levels during biofilm development by RNA-Seq to establish transcriptome profiles of free vs. biofilm *S. cerevisiae* cells and to obtain a better understanding of the regulatory mechanisms of biofilm development. We identified critical genes and metabolic pathways that were significantly up- or down-regulated during biofilm development. When comparing gene expression levels observed in the three phases (the attachment period, the sessile-growth, and biofilm-maturation periods) with those observed in free cells, we found that most gene expression differences occurred during the attachment period. Analysis of signaling pathways mediated by the differentially expressed genes identified in this study showed that biofilms are regulated by the MAPK and cAMP-PKA pathways. To further confirm the potential regulatory relationships between biofilms and other genes, in-depth analysis was performed, which showed that the *Phd1*, *Ash1*, *Mga1*, and *Sok2* genes may be involved with cAMP-PKA/MAPK in regulating biofilm genesis (Figure 5). In addition, we found that during the attachment period of biofilms, the expression of gluconeogenesis pathway genes was up-regulated, and glycolysis was restricted. However, the expression of gluconeogenesis pathway genes was down-regulated and glycolysis was up-regulated during the biofilm maturation period, suggesting that glycolysis/gluconeogenesis is needed for biofilm formation. Moreover, we also validated the expression of previously implicated biofilm genes and identified novel genes in our biofilms. The present work serves as a basis for future studies examining the complex network systems that regulate *S. cerevisiae* biofilm formation and maintenance, and more work is necessary to elucidate the exact role of these genes in *S. cerevisiae* biofilm development.

### Conflict of interest statement

The authors declare that the research was conducted in the absence of any commercial or financial relationships that could be construed as a potential conflict of interest.

## Supplementary material

Table S1 **GO analysis of FCP-vs.-AP**.

Table S2 **GO analysis of FCP-vs.-SGP**.

Table S3 **GO analysis of FCP-vs.-BMP**.

Table S4 **KEGG analysis of FCP-vs.-BMP**.

Table S5 **KEGG analysis of FCP-vs.-BMP**.

Table S6 **KEGG analysis of FCP-vs.-BMP**.

Table S7 **Real - time quantitative PCR data**.
