# Supplementary material for: Involvement of glycolysis/gluconeogenesis and signaling regulatory pathways in Saccharomyces cerevisiae biofilms during fermentation
Source: Front Microbiol. 2015 Feb 23;6:139. doi: 10.3389/fmicb.2015.00139 (PMC4337339; doi:10.3389/fmicb.2015.00139)
Supplement: Table S7 — Real - time quantitative PCR data. [file DataSheet7.DOCX]

**Table S7. RT-PCR data**

Forward (F) and reverse (R) primer sequences for quantitative reverse transcription-PCR (qRT-PCR) analysis of selected genes in *S. cerevisiae*. The presented data for qRT-PCR are means ± SEM of three independent experiments.

| **Gene ID** | **Gene name** | **Primer sequences** | **Fold changes of gene expression in biofilm cells vs. free cells** | | | | | |
| --- | --- | --- | --- | --- | --- | --- | --- | --- |
|  |  |  | **The attachment period** | | **The sessile-growth** | | **Biofilm-maturation period** | |
|  |  |  | **RNA-Seq** | **RT-PCR** | **RNA-Seq** | **RT-PCR** | **RNA-Seq** | **RT-PCR** |
|  | *18S* (reference gene) | F: TGTGCTGGCGATGGTTCA  R: TGCCTTCCTTGGATGTGGTA | 0.9 | 1 | 1 | 1 | 10.3 | 1 |
| YIR019C | *Flo11* | F: CCCTGTCACGACGGCTATT  R: TTGGCACCATTTGAACCTG | 6.8 | 4.0±0.4 | 5.1 | 4.5±0.5 | 18.3 | 14.3±0.5 |
| YKR097W | *Pck1* | F: AAACCACTTTATCCGCAGACC  R: TTGGCGTAACAACCACCTTC | 554.6 | 420.6±4.8 | 1.8 | 2.0±0.6 | 1.1 | 0.9±0.3 |
| YLR377C | *Fbp1* | F: ATTGACATTGGGTGATGGAGTT  R: TCTGCTTGGGGTTGTTTGAC | 233.6 | 340.7±5.6 | 1.1 | 0.4±0.3 | 1.2 | 1.7±0.3 |
| YFL014W | *Hsp12* | F: GACAAGGTCGCTGGTAAGGTT  R: GGCATCGTTCAACTTGGACTT | 181.0 | 215.6±4.3 | 48.9 | 44.4±0.4 | 12.7 | 21.2±0.3 |
| YGL035C | *Mig1* | F: TCTCCCAAAACGATGGCTAA  R: ACTATGGCTATTGCTCAACGAA | 25.6 | 25.4±0.7 | 1.9 | 1.7±0.4 | 1.0 | 1.2±0.2 |
| YJL089W | *Sip4* | F: CCAAGAGGAAATATGGCAGGTC  R: CAGTGATTGCGTTAGAGGAGC | 27.0 | 31.6±0.3 | 1.9 | 2.1±0.2 | 2.5 | 2.0±0.6 |
